# Supplementary material for: Spatial distribution and determinants of HIV prevalence among adults in urban Ethiopia: Findings from the Ethiopia Population-based HIV Impact Assessment Survey (2017–2018)
Source: PLoS One. 2022 Jul 12;17(7):e0271221. doi: 10.1371/journal.pone.0271221 (PMC9491827; doi:10.1371/journal.pone.0271221)
Supplement: Attachment — Submitted filename: Reviewer Recommendation and Comments for Manuscript Number PONE.pdf [file pone.0271221.s002.pdf]

Reviewer Recommendation and Comments for Manuscript Number PONE-D-21-34281

Manuscript Title: Spatial distribution and determinants of HIV prevalence among adults in urban Ethiopia: Findings from the Ethiopia Population-based HIV Impact Assessment Survey (2017–2018)

Original Submission

Reviewer: Masimalai Palaniyandi

- The scope of the study is well designed to delineate hotspot HIV transmission risk.
- The study result cloud be used to control and management of the HIV infection in the vulnerable community through proper intervention measures target the gender and affected age groups with efforts of public health officials and national health programmers
- Random Sampling methods for field data collection of HIV epidemiological information in the selected house hold, and geo-coordinates of the house hold location are depicted on map, using GPS.
- Geo-Spatial analysis was applied significantly
- Moran I geo-statistical analysis measure of spatial autocorrelation in area data, explaining the degree of dependence between values of a variable at different geographic locations.
- Geographically weighted regression (GWR) analysis was used to establish the relationship of covariates.
- The interpolation of spatial prediction of HIV infection using Kriging methods, and results reflect the real situation of the ground.
- Recommended for the publication
