# Supplementary material for: Spatial distribution and determinants of HIV prevalence among adults in urban Ethiopia: Findings from the Ethiopia Population-based HIV Impact Assessment Survey (2017–2018)
Source: PLoS One. 2022 Jul 12;17(7):e0271221. doi: 10.1371/journal.pone.0271221 (PMC9491827; doi:10.1371/journal.pone.0271221)
Supplement: Attachment — Submitted filename: A rebuttal letter .docx [file pone.0271221.s003.docx]

PONE-D-21-34281

Spatial distribution and determinants of HIV prevalence among adults in urban Ethiopia: Findings from the Ethiopia Population-based HIV Impact Assessment Survey (2017–2018)

PLOS ONE

Dear Dr. Argefa,

Thank you for submitting your manuscript to PLOS ONE. After careful consideration, we feel that it has merit but does not fully meet PLOS ONE’s publication criteria as it currently stands. Therefore, we invite you to submit a revised version of the manuscript that addresses the points raised during the review process.

Please submit your revised manuscript by Feb 13 2022 11:59PM. If you will need more time than this to complete your revisions, please reply to this message or contact the journal office at [plosone@plos.org](mailto:plosone@plos.org). When you're ready to submit your revision, log on to <https://www.editorialmanager.com/pone/> and select the 'Submissions Needing Revision' folder to locate your manuscript file.

Please include the following items when submitting your revised manuscript:

- A rebuttal letter that responds to each point raised by the academic editor and reviewer(s). You should upload this letter as a separate file labeled 'Response to Reviewers'.
- A marked-up copy of your manuscript that highlights changes made to the original version. You should upload this as a separate file labeled 'Revised Manuscript with Track Changes'.
- An unmarked version of your revised paper without tracked changes. You should upload this as a separate file labeled 'Manuscript'.

If you would like to make changes to your financial disclosure, please include your updated statement in your cover letter. Guidelines for resubmitting your figure files are available below the reviewer comments at the end of this letter.

If applicable, we recommend that you deposit your laboratory protocols in [protocols.io](http://protocols.io/) to enhance the reproducibility of your results. Protocols.io assigns your protocol its own identifier (DOI) so that it can be cited independently in the future. For instructions see: <https://journals.plos.org/plosone/s/submission-guidelines#loc-laboratory-protocols>. Additionally, PLOS ONE offers an option for publishing peer-reviewed Lab Protocol articles, which describe protocols hosted on [protocols.io](http://protocols.io/). Read more information on sharing protocols at <https://plos.org/protocols?utm_medium=editorial-email&utm_source=authorletters&utm_campaign=protocols>.

We look forward to receiving your revised manuscript.

Kind regards,

Mona Pathak, PhD
Academic Editor
PLOS ONE

Journal Requirements:

When submitting your revision, we need you to address these additional requirements.

1. Please ensure that your manuscript meets PLOS ONE's style requirements, including those for file naming. The PLOS ONE style templates can be found at

<https://journals.plos.org/plosone/s/file?id=wjVg/PLOSOne_formatting_sample_main_body.pdf> and

<https://journals.plos.org/plosone/s/file?id=ba62/PLOSOne_formatting_sample_title_authors_affiliations.pdf>

**Authors’ Response**: Double-checked to ensure that the manuscript meets PLOS ONE's style requirements, including the file naming. As suggested, a thorough copy-edit of the manuscript has been made to improve clarity.

2. Thank you for stating the following in the Acknowledgments Section of your manuscript:

“This project has been supported by the U.S. President’s Emergency Plan for AIDS Relief (PEPFAR) through the U.S. Centers for Disease Control and Prevention (CDC) under the terms of cooperative agreement #U2GGH001226. The findings and conclusions in this report are those of the authors and do not necessarily represent the official position of the funding agencies. Conflict of interest.”

We note that you have provided additional information within the Acknowledgements Section that is not currently declared in your Funding Statement. Please note that funding information should not appear in the Acknowledgments section or other areas of your manuscript. We will only publish funding information present in the Funding Statement section of the online submission form.

Please remove any funding-related text from the manuscript and let us know how you would like to update your Funding Statement. Currently, your Funding Statement reads as follows:

 “The author(s) received no specific funding for this work.”

Please include your amended statements within your cover letter; we will change the online submission form on your behalf.

**Authors’ Response**: As it was already indicated in the funding statement, the author(s) received no specific funding for this work. We have opted to removing the funding information form the manuscript.

3. We note that you have stated that you will provide repository information for your data at acceptance. Should your manuscript be accepted for publication, we will hold it until you provide the relevant accession numbers or DOIs necessary to access your data. If you wish to make changes to your Data Availability statement, please describe these changes in your cover letter and we will update your Data Availability statement to reflect the information you provide.

**Authors’ Response**: Data is availability at the following link

<https://phia-data.icap.columbia.edu/datasets?country_id=12>

4. We note that Figure 1, 2, 3 and 4 in your submission contain map images which may be copyrighted. All PLOS content is published under the Creative Commons Attribution License (CC BY 4.0), which means that the manuscript, images, and Supporting Information files will be freely available online, and any third party is permitted to access, download, copy, distribute, and use these materials in any way, even commercially, with proper attribution. For these reasons, we cannot publish previously copyrighted maps or satellite images created using proprietary data, such as Google software (Google Maps, Street View, and Earth). For more information, see our copyright guidelines: <http://journals.plos.org/plosone/s/licenses-and-copyright>.

We require you to either (1) present written permission from the copyright holder to publish these figures specifically under the CC BY 4.0 license, or (2) remove the figures from your submission:

 a. You may seek permission from the original copyright holder of Figure 1, 2, 3 and 4 to publish the content specifically under the CC BY 4.0 license.

We recommend that you contact the original copyright holder with the Content Permission Form (<http://journals.plos.org/plosone/s/file?id=7c09/content-permission-form.pdf>) and the following text:

“I request permission for the open-access journal PLOS ONE to publish XXX under the Creative Commons Attribution License (CCAL) CC BY 4.0 (<http://creativecommons.org/licenses/by/4.0/>). Please be aware that this license allows unrestricted use and distribution, even commercially, by third parties. Please reply and provide explicit written permission to publish XXX under a CC BY license and complete the attached form.”

Please upload the completed Content Permission Form or other proof of granted permissions as an "Other" file with your submission.

In the figure caption of the copyrighted figure, please include the following text: “Reprinted from [ref] under a CC BY license, with permission from [name of publisher], original copyright [original copyright year].”

 b. If you are unable to obtain permission from the original copyright holder to publish these figures under the CC BY 4.0 license or if the copyright holder’s requirements are incompatible with the CC BY 4.0 license, please either i) remove the figure or ii) supply a replacement figure that complies with the CC BY 4.0 license. Please check copyright information on all replacement figures and update the figure caption with source information. If applicable, please specify in the figure caption text when a figure is similar but not identical to the original image and is therefore for illustrative purposes only.

The following resources for replacing copyrighted map figures may be helpful:

USGS National Map Viewer (public domain): <http://viewer.nationalmap.gov/viewer/>

The Gateway to Astronaut Photography of Earth (public domain): <http://eol.jsc.nasa.gov/sseop/clickmap/>

Maps at the CIA (public domain): <https://www.cia.gov/library/publications/the-world-factbook/index.html> and <https://www.cia.gov/library/publications/cia-maps-publications/index.html>

NASA Earth Observatory (public domain): <http://earthobservatory.nasa.gov/>

Landsat: <http://landsat.visibleearth.nasa.gov/>

USGS EROS (Earth Resources Observatory and Science (EROS) Center) (public domain): [http://eros.usgs.gov/#](http://eros.usgs.gov/)

Natural Earth (public domain): <http://www.naturalearthdata.com/>

5. Please ensure that you refer to Figure 1 and 2 in your text as, if accepted, production will need this reference to link the reader to the figure.

**Authors’ Response**: The Ethiopia shapefiles are public domains available at <https://africaopendata.org/dataset/ethiopia-shapefiles> and the EPHIA geospatial date sets are available at <https://phia-data.icap.columbia.edu/login>. There is no need for copyright information on the figures that the author generated.

[Note: HTML markup is below. Please do not edit.]

Reviewers' comments:

Reviewer's Responses to Questions

**Comments to the Author**

1. Is the manuscript technically sound, and do the data support the conclusions?

The manuscript must describe a technically sound piece of scientific research with data that supports the conclusions. Experiments must have been conducted rigorously, with appropriate controls, replication, and sample sizes. The conclusions must be drawn appropriately based on the data presented.

Reviewer #1: Yes

Reviewer #2: No

**Authors’ Response**: As a review of the conclusion has been made to improve clarity.

2. Has the statistical analysis been performed appropriately and rigorously?

Reviewer #1: Yes

Reviewer #2: No

**Authors’ Response**: A review of the statistical analysis has been made to improve clarity.

3. Have the authors made all data underlying the findings in their manuscript fully available?

The [PLOS Data policy](http://www.plosone.org/static/policies.action#sharing) requires authors to make all data underlying the findings described in their manuscript fully available without restriction, with rare exception (please refer to the Data Availability Statement in the manuscript PDF file). The data should be provided as part of the manuscript or its supporting information, or deposited to a public repository. For example, in addition to summary statistics, the data points behind means, medians and variance measures should be available. If there are restrictions on publicly sharing data—e.g. participant privacy or use of data from a third party—those must be specified.

Reviewer #1: Yes

Reviewer #2: No

**Authors’ Response**: The data is available at <https://phia-data.icap.columbia.edu/datasets?country_id=12> without restriction.

4. Is the manuscript presented in an intelligible fashion and written in standard English?

PLOS ONE does not copyedit accepted manuscripts, so the language in submitted articles must be clear, correct, and unambiguous. Any typographical or grammatical errors should be corrected at revision, so please note any specific errors here.

Reviewer #1: Yes

Reviewer #2: Yes

5. Review Comments to the Author

Please use the space provided to explain your answers to the questions above. You may also include additional comments for the author, including concerns about dual publication, research ethics, or publication ethics. (Please upload your review as an attachment if it exceeds 20,000 characters)

Reviewer #1: 
¬ The scope of the study is well designed to delineate hotspot HIV transmission risk.
¬ The study result cloud be used to control and management of the HIV infection in the vulnerable community through proper intervention measures target the gender and affected age groups with efforts of public health officials and national health programmers
¬ Random Sampling methods for field data collection of HIV epidemiological information in the selected house hold, and geo-coordinates of the house hold location are depicted on map, using GPS.
¬ Geo-Spatial analysis was applied significantly
¬ Moran I geo-statistical analysis measure of spatial autocorrelation in area data, explaining the degree of dependence between values of a variable at different geographic locations.
¬ Geographically weighted regression (GWR) analysis was used to establish the relationship of covariates.
¬ The interpolation of spatial prediction of HIV infection using Kriging methods, and results reflect the real situation of the ground.

Reviewer #2: Please find my comments and suggestions below. I have mainly focused on the spatial analysis aspects of this study and please read my suggestions below.

Study variables and measurement (Lines 87-98)
Please clearly define your outcome variable. Please define the geo-linked HIV prevalence, geo-linked to what geographical level? It is not clearly described how the enumeration areas (EAs) are related to “towns” Does each enumeration area consist of one town? Or do you need to aggregate several EAs to the town level?
When you define the prevalence of HIV, it is not clear what a cluster means. Does “cluster” refer to an EA or a town? When you are mentioning that you have calculated the weighted HIV prevalence in each town, but you did not describe the weighting method.

**Authors’ Response:** Corrected.

In Figure 2 you are presenting a prevalence map, but the area boundaries are not defined. Are those provinces in Ethiopia?

**Authors’ Response**: Corrected. Area boundaries indicate administrative regions in Ethiopia.

Additionally, as you have mentioned just a small portion of EAs could be sampled from “Somali” district, and for that reason, you should change the map to include a small portion of that district. Figure 2 implies that HIV prevalence is low in Somali district, but we do not know because the population was not included in the EPHIA survey.

**Authors’ Response**: Yes, as described in the limitation section, Somali had a relatively small number of HIV-positive individuals, which may raise questions related to accuracy of some estimates in these regions.

Line 91 – Please state that the outcome variable for your regression model (OLS) was the weighted HIV prevalence in each town.

**Authors’ Response**: Corrected

Line 92 – “Study variables” is a confusing term, because study variables could include the outcome (e.g., dependent) variable and the predictor (e.g., independent) variables. You should use “predictor” or “independent” variables instead of study variables.

**Authors’ Response**: Corrected

Analysis (Lines 139-190)
The hotspot analysis (Getis-Ord Gi*statistic) is not a spatial interpolation method. Moreover, you did not present any local clustering (hot spot) analysis. Please describe in detail your Kriging method, because there is more than one Kriging method that you can use. Kriging is a spatial interpolation method and you can not identify statistically significant clusters of high or low HIV prevalence based on Kriging analysis. You might confuse Kriging with the Getis-Ord Gi* statistic or the Local Moran’s I method where you can identify high or low (if you use Getis-Ord Gi*) and high-high, low-low, low-high, and high-low (if you use Local Moran’s I) HIV prevalence clusters, but you did not present any of those results.
You can read my previous papers where I have used and described various spatial epidemiology methods:
<https://journals.plos.org/plosone/article?id=10.1371/journal.pone.0235291>
<https://bmcinfectdis.biomedcentral.com/articles/10.1186/s12879-015-1106-6>

**Authors’ Response**: Revisions have been made in the manuscript

Line 148 - Global Moran’s I statistic evaluates the extent of spatial heterogeneity. You should define at what distance band did you observe the highest Moran's I value (see my papers for the methods). Please also define what type of conceptualization parameter did you use, because you can use more than one (e.g., zone of indifference, inverse distance, etc.). I think that you should use and present local Moran’s I analysis to identify local HIV clusters.

**Authors’ Response**: Edits have been made in the manuscript

Line 168 – Please define your GWR model. What type of spatial weights matrix did you use? What type of GRW did you use, spatial lag model or spatial error model?
You can read about them at:
<https://s4.ad.brown.edu/resources/tutorial/modul2/geoda3final.pdf>

**Authors’ Response**: Specified geographically weighted regression in the manuscript.

Thank you!

6. PLOS authors have the option to publish the peer review history of their article ([what does this mean?](https://journals.plos.org/plosone/s/editorial-and-peer-review-process#loc-peer-review-history)). If published, this will include your full peer review and any attached files.

If you choose “no”, your identity will remain anonymous but your review may still be made public.

**Do you want your identity to be public for this peer review?** For information about this choice, including consent withdrawal, please see our [Privacy Policy](https://www.plos.org/privacy-policy).

Reviewer #1: **Yes:** Masimalai Palaniyandi., M.Sc.,M.Tech.,Ph.D.,

Reviewer #2: **Yes:** Csaba Varga

[NOTE: If reviewer comments were submitted as an attachment file, they will be attached to this email and accessible via the submission site. Please log into your account, locate the manuscript record, and check for the action link "View Attachments". If this link does not appear, there are no attachment files.]

While revising your submission, please upload your figure files to the Preflight Analysis and Conversion Engine (PACE) digital diagnostic tool, <https://pacev2.apexcovantage.com/>. PACE helps ensure that figures meet PLOS requirements. To use PACE, you must first register as a user. Registration is free. Then, login and navigate to the UPLOAD tab, where you will find detailed instructions on how to use the tool. If you encounter any issues or have any questions when using PACE, please email PLOS at [figures@plos.org](mailto:figures@plos.org). Please note that Supporting Information files do not need this step.

*In compliance with data protection regulations, you may request that we remove your personal registration details at any time.* [*(Remove my information/details)*](https://www.editorialmanager.com/pone/login.asp?a=r)*. Please contact the publication office if you have any questions.*
